# Competition-Based Model of Pheromone Component Ratio Detection in the Moth

**DOI:** 10.1371/journal.pone.0016308

**Published:** 2011-02-16

**Authors:** Andrei Zavada, Christopher L. Buckley, Dominique Martinez, Jean-Pierre Rospars, Thomas Nowotny

**Affiliations:** 1 Informatics, University of Sussex, Brighton, United Kingdom; 2 CORTEX Team - LORIA, Vandoeuvre-lès-Nancy, France; 3 UMR 1272 Physiologie de l'insecte, INRA, Versailles, France; Mount Sinai School of Medicine, United States of America

## Abstract

For some moth species, especially those closely interrelated and sympatric, recognizing a specific pheromone component concentration ratio is essential for males to successfully locate conspecific females. We propose and determine the properties of a minimalist competition-based feed-forward neuronal model capable of detecting a certain ratio of pheromone components independently of overall concentration. This model represents an elementary recognition unit for the ratio of binary mixtures which we propose is entirely contained in the macroglomerular complex (MGC) of the male moth. A set of such units, along with projection neurons (PNs), can provide the input to higher brain centres. We found that (1) accuracy is mainly achieved by maintaining a certain ratio of connection strengths between olfactory receptor neurons (ORN) and local neurons (LN), much less by properties of the interconnections between the competing LNs proper. An exception to this rule is that it is beneficial if connections between generalist LNs (i.e. excited by either pheromone component) and specialist LNs (i.e. excited by one component only) have the same strength as the reciprocal specialist to generalist connections. (2) successful ratio recognition is achieved using latency-to-first-spike in the LN populations which, in contrast to expectations with a population rate code, leads to a broadening of responses for higher overall concentrations consistent with experimental observations. (3) when longer durations of the competition between LNs were observed it did not lead to higher recognition accuracy.

## Introduction

In moths, males are attracted to females by means of sexual pheromones, which are often composed of a blend of two or more components. Pheromone signalling serves as an interspecific barrier which, along with temporal and geographical separation of the populations, arises from and facilitates the process of speciation. In some species, *e.g.*, *Spodoptera littoralis*, the secondary component modulates the behavioural response to the primary component but is not strictly necessary to attract the male [1], [2]. Other species maintain a blend of two (*e.g.*, *Manduca sexta*: [3]) or more components, as in *Agrotis segetum* [4], *Heliothis/Helicoverpa* species [5], [6] and *Ostrinia* species [7]. In the latter two cases, different species were shown to use subsets of the same group of chemical compounds in their pheromone blend, but in different combinations, roles and/or concentration ratios. In these cases, in order to be effective in the wild, relevant components must all be present in the pheromone plume in certain proportions, and recognising the correct ratio of pheromone component concentrations becomes critical for the mating success of the male.

Previous modelling work of the pheromone subsystem goes back to early 1990's. Linster *et al* [8] were successful in replicating the various different response patterns observed experimentally in *Manduca sexta* projection neurons (PNs) by varying the number of excitatory and inhibitory local neurons (LNs) in an abstract model of the male moth Macroglomerular Complex (MGC) where all connections between pheromone-sensitive ORNs, LNs and PNs are made. They, however, refrained from identifying patterns which may indicate successful ratio discrimination, arguing that “it is not known whether the detection of a precise ratio is achieved at the level of the glomerulus or at higher olfactory centers”. Although whether the ratio detection occurs at the MGC or at a later stage (*e.g.*, in the mushroom bodies), is generally species-dependent [9], there is evidence [10] that, in *Agrotis segetum*, PNs relay both component-specific as well as blend-specific signals to higher brain centres. This suggests that at least partially, ratio recognition takes place within the LN-PN network in the MGC.

Extending their earlier work, Linster *et al* [11] considered a binary blend of major and minor components and suggested that component ratio detection is signalled by the presence of oscillations rather than the overall firing rate in the PN population in the MGC. In another work they refined their models by introducing several physiological constraints [12]. While oscillations have been observed in the MGC of *M. sexta* [13] as well as in the general olfactory subsystem of locusts [14], recent work in our group indicates that oscillations do not seem to be present in the pheromone subsystem of *Agrotis ipsilon* (unpublished data; A.Chaffiol, pers. comm.). Similarly, while oscillations have been found in the locust, suppressing them with picrotoxin injections does not fully abolish odour recognition [15]. It also remains open how the downstream brain centres would estimate the spectral power of the PN output if the presence or absence of oscillations was the main carrier of information.

Similarly to Linster and colleagues, Getz & Lutz [16] used a detailed recurrent network model of LN and PN populations to explain the emergence of odour specific response patterns that allowed odour quality recognition for varying concentrations. Subsequent works of Bazhenov [17], [18] put forward a model for the locust antennal lobe including both fast oscillations and slow patterning.

The slow patterning of odour responses and the spatio-temporal coding were incorporated into the theoretical framework of “winnerless competition” [19], [20] to explain discrimination, and possibly learning, of a variety of odours in the general olfactory system. In this framework, the presence of synchronous oscillations is interpreted as a signal of the presence of odour input and slow patterning as an odour's distinguishing characteristic. More recently, Kwok [21] explored the behaviour of a model with direct ORN-PN and feedback LN-PN connections, and described the temporal evolution of the model state and related it to winnerless competition. Individual odours as well as odour blends in Kwok's model have distinct spatio-temporal representations. However, while the combinatorial explosion of spatio-temporal patterns is a distinctive advantage for coding the vast space of relevant odour inputs in the general olfactory system, it is difficult to identify a role for spatio-temporal patterns in the pheromone subsystem where the components are few and known beforehand, and only a certain fixed target ratio is biologically relevant.

### Competition prerequisites

**Figure 1:**
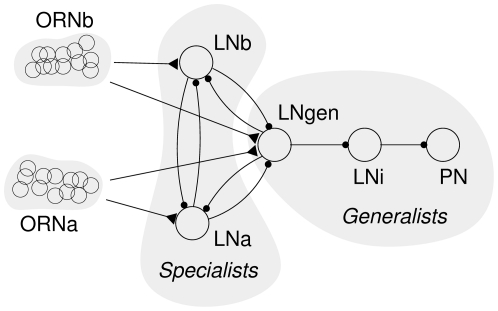
The structure of the elementary pheromone component ratio recognition unit in the MGC. Two groups of ORNs (ORNa and ORNb), each tuned to a specific individual pheromone component, converge onto ipsilateral specialist LNs (LNa and LNb) and onto a generalist LN (LNc). All LNs are interconnected via inhibitory synapses. The response of the model is observed at the intermediary LNi and the PN.

In this paper we study minimalistic models of competition between LNs in the MGC and how they allow recognising a specific pheromone component ratio across a wide range of pheromone concentrations. The simplest model, “an elementary ratio recognition unit” shown in fig. 1, is an essentially feed-forward (*i.e.*, lacking feedback connections such as from PNs to LNs or LNs to ORNs) network that is capable of recognising a target ratio of a blend of two pheromone components. Over a range of one order of magnitude of ORN response frequency, the unit produces a discrete ‘yes/no’ signal, in the form of a steady spike train or quiescence, identifying the matching target component ratio determined by its intrinsic properties. If the ORN response is logarithmic in the concentration, this corresponds to an operating range of 7 orders of magnitude in pheromone concentration. The model uses conductance based, Hodgkin–Huxley type models for LNs and PNs [22], [23] and first order synapses [24], [25].

While not biased towards a particular coding paradigm by design, we demonstrate below that decision-making in this model is entirely reduced to the competition between LNs in terms of latency to the first spike: the LN that spikes first becomes the effective ‘winner’ even though it may receive less excitation on average during the course of stimulation than other LNs. The noisy nature of the ORN signalling, instantiated by independent Poisson processes in our model, and the different initial conditions of the LNs can thus lead to a significant number of errors in recognising the correct ratio. Accordingly, the main factor affecting the accuracy and performance of this model is the ORN-LN convergence rate which controls the signal to noise ratio in the ORN signal.

We then extend our model in two different ways. First, we consider a population of the discussed elementary recognition units and then a population unit, where the individual LNs are replaced with more realistic populations. We find that the population models allow for a more gradual “rate” competition and perform better than the elementary unit. We also tested the different models within the framework of approximately equivalent rate models (see Text S1).

## Materials and Methods

### The basic model topology and units

Our basic model of the male MGC for a species utilizing a blend of two pheromone components can be schematically represented as shown in fig. 1.

#### Olfactory Receptor Neurons

We consider two groups of ORNs, each composed of cells that are narrowly tuned to respond very specifically to one of the two components of the female sex pheromone. The number of ORNs of each type is, as we argue below, an important factor affecting the accuracy of ratio detection. The spiking response of ORNs in the presence of pheromone has been shown to follow Poisson statistics with stimulus dependent rate over a wide range of stimulus intensities [26]. The firing rate *λ* of the Poissonian ORN spike trains of a given ORN population is approximately proportional to the logarithm of the concentration of the pheromone component it responds to. Specifically, Kaissling [27] gives a dose–response curve for *Antheraea polyphemus* where a flow of 10^−8^ µg/sec of (E,Z)-6,11-hexadecadienyl acetate causes ORNs to fire at approximately 10 Hz. With the pheromone concentrations up to 10^−2^ µg/sec, the unadapted (*i.e.*, not previously exposed to the pheromone) ORNs remain sensitive and increase the firing rate to 300 Hz in a fairly linear fashion. The range of firing rates of the ORNs in our study is 10–200 Hz. In terms of pheromone concentrations, this approximately corresponds to 10^−8^–10^−3^ µg/sec [27].

Our ORNs have a resting potential of −60 mV and emit 80 mV ‘durationless’ (*i.e.*, lasting for the current value of the integration time step) impulses.

As the summation of individual Poisson processes is still a Poisson process, further optimisation has been applied in which a group of ORNs is emulated by a single “compound” Poisson model neuron. The compound ORN has frequency *λ* = *λ*_s_ *N*_ORN_, where *λ*_s_ is the frequency of a single ORN and *N*_ORN_ is the size of the represented ORN population. The high spiking rate generated by these compound ORNs results in a significant probability of multiple spikes occurring within a single time step Δ*t*. To account for this we modified the standard first order synapse model by Destexhe et al [25]:
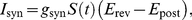
(1)

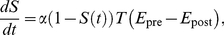
(2)where *α* and *β* are the rates of neurotransmitter release and decay, respectively, and *T* is a step function which equals unity for *t*_release_ after each pre-synaptic spike to simulate the finite duration of transmitter release following a pre-synaptic spike. *E*_pre_ and *E*_post_ are the pre- and postsynaptic membrane potentials and *E*_rev_ the reversal potential of the synapse. The release of transmitter due to spikes within a single time step and across time steps within *t*_release_ of each other is summed according to
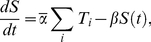
(3)where *T_i_* = *q* in the interval [*t_i_*, *t_i_*+*t*_release_] if *q* spikes occurred at time *t_i_* and the sum is over all relevant time steps *t_i_* in [*t*−*t*_release_, *t*]. *α* is determined such that for a single spike the resulting maximal synaptic activation *S* is the same in both the ORN population and compound ORN descriptions. This is the case for 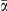 = 0.556 *α*.

#### Convergence rate of ORNs to LNs

In male *Manduca sexta* antenna, there are 42,848±3,374 *sensilla trichodea*, each bearing on average two pheromone-sensitive ORNs [28], with a total of about 85,700 ORNs. These project to the MGC to form synapses with LNs, of which Matsumoto & Hildebrand [29] distinguish several types. The authors identified a class of LNs only found in males and responding to pheromone substances as ‘Type II’ LNs and estimated the proportion of Type II LNs, in a sample of over 1000 cells stained, to be about 25% (namely, (86+68)/(260+86+68)). In a later work Homberg *et al* [30] confirmed LNs of *M. sexta* to have their somata grouped in three clusters on the periphery of the AL with a total cell count of 991 in the lateral cell group, 16, in the anterior, and 218 in the medial cell group (of these, only the latter is sexually dimorphic having 186 cells in females). The total number of LN somata in all cell groups is thus 1225, which includes LNs both pheromone-sensitive as well as those involved in the general olfactory system. Ultimately, from the observation that Matsumoto & Hildebrand [29] “have not detected any pattern in the locations of the cell bodies of the various types of neurons within the lateral and medial clusters,” the closest gross estimate for the number of pheromone-sensitive LNs is thus 25% of 1225, or some 300 cells.

Given the fact that ORN axons arborise in the glomeruli, and hence, form synapses with more than a single LN in the MGC, the actual convergence ratio can be larger than 85,700/300, or upwards of 285∶1.

#### Local Neurons and the competition principle

We consider two ORN populations, ORNa amd ORNb, which are each selectively responsive to one of two pheromone components, ORNa to component A and ORNb to component B. LNs receive excitatory inputs from one of the two ORN populations (LNa and LNb, sensitive to pheromone components A and B, respectively) or from both (LNc). According to their innervation, LNa and LNb are termed ‘specialist’ LNs, and LNc is a ‘generalist’ LN. Both response types of LNs have been found experimentally [31], [5]. All LNs are interconnected through inhibitory synapses, in agreement with the findings of numerous studies [31], [32], [33], [34], [35] showing that connections between LNs in the glomeruli are inhibitory (GABAergic), though see [36]. When a pheromone blend is presented which has a component ratio sufficiently close to the target value, the ORNs will activate their LN targets and the competition between LNs, mediated by the local inhibitory connections will, given appropriate inter-LN synaptic strengths, cause the generalist LN to activate most and inhibit the specialist LNs. This in turn will inhibit the LNi, disinhibiting the PN and causing it to spike. Conversely, when the component ratio is deviating from the target value, one of the specialist LNs will suppress the other specialist LN as well as the generalist LN and prevent them from spiking. The LNi will continue spiking and there will be no response in the PN. In this way the system implements a winner-takes-all scenario where one of the specialist LNs ‘wins’ if its own component dominates the blend and the generalist LN wins if the blend has the correct target ratio. The generalist LNc is the exit point for the ‘ratio detected’ signal (to be picked up by a projection neuron and delivered further downstream to higher brain centres).

#### Projection Neurons

The spiking response elicited by the stimulation at LNc will be dependent on the intensity of the stimulation, *i.e.*, the pheromone component concentration. Since our concern is ratio recognition *irrespective of* component concentration, we utilise the mechanism of disinhibition in the intermediate neuron LNi and the PN, in order to achieve a discrete ‘yes/no’ response as model output. This disinhibition pathway also allows to adhere to the biological principle that typically only one transmitter is used in any given neuron — the competing LNs are inhibitory whereas the PN is excitatory. The latter two are also spontaneously active (implemented *via* the injection of a constant current of 0.1 nA in the model). LN-LNi and LNi-PN synapses are inhibitory. One previous work in which this same mechanism has been studied in an AL model at the PN stage, is by Av-Ron and colleagues [37], [38], who also substantiate a biological relevance of disinhibition occurring in PNs.

All LNs and the PN in the model are described by Hodgkin–Huxley model neurons [22], [23] connected by generic synapses following Destexhe *et al* [24] amended as explained above; for details and equations, see Text S1. While there have been recent advances on Ca^2+^ currents in the LNs of the cockroach [39], [40], [41] no complete and detailed voltage clamp data set or existing specific Hodgkin-Huxley models for moth AL LNs has been accessible to us. We, therefore, chose the popular, excitability type 1 Hodgkin-Huxley model neuron of Traub and Miles [22], [23] for the LNs and the PN. This neuron model allows a wide range of gradual changes in spike frequency which seemed appropriate for our study. The model has also already been used extensively in previous models of an insect AL [17], [18] and seems to be well suited for allowing the competition dynamics that we seek to evaluate.

### Simulation of the Hodgkin–Huxley neurons

All ordinary differential equations for Hodgkin–Huxley neurons and corresponding rate models were integrated using a 6-5-order Runge–Kutta algorithm adapted from Press *et al* [42], with adaptive time step Δ*t* confined to 10^−6^–0.5 ms and accuracy goal *ε* = 10^−12^.

The code of our simulations comes in two parts, a general simulation engine “cnrun”, available at sourceforge.net/projects/cnrun or johnhommer.com/academic/cnrun, and model descriptions in NeuroML, available at johnhommer.com/academic/ratiocoding.

#### Rate-based reduction of the Hodgkin–Huxley model

Inherent in the competition approach is the issue that the timing of phasic events (spikes) may not reflect the true input ratio due to the stochasticity of the ORNs, and a resulting non-vanishing probability of locking the state of competing LNs into a ‘wrong state.’ To assess the relevance of spikes we studied the ratio recognition capability of an otherwise identical model built with a rate approximation of the Hodgkin–Huxley neurons (for the derivation of this model from the conductance-based equations, see Text S1). In the rate model, the LNc spiking rate averaged over the duration of each stimulus presentation replaces the spike density function in the response matrix (see below).

### Experimental protocol and trial cost function

We tested the model systematically with 100 different component concentration combinations, using firing rates *λ*_a_, *λ*_b_ for the two populations of ORNs in increments of *λ_i_* = 1.3 *λ_i_*_−1_ for *i* = 0…9, starting with *λ*_0_ = 0.01 ms^−1^ which allows us to cover a range of 10 to 100 Hz for ORN firing, a realistic range. Each stimulus frequency pair was presented for 250 ms, with 250 ms between presentations which was inspired by the ability of moths to resolve pulsed odor trains at this order of frequency.

**Figure 2:**
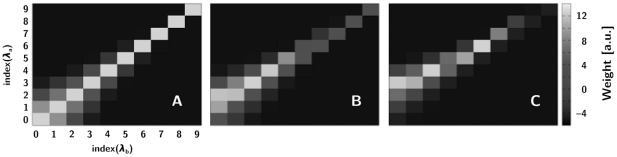
The target profile matrix for the conductance-based setup, with the x axis referring to λ_a_^i^ = λ_0_×1.3^i^, i = 0,…,9, the y axis to λ_b_^j^ = λ_0_×1.3^j^, j =  0,…,9 and λ_0_ = 0.01 ms^−1^. Individual weights are 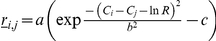 for the target ratios *R* of 1∶1 (panel **A**), 1∶3 (**B**) and 1∶9 (**C**), where *C_i_* = 2×1.3*^i^* and *a* = 18, *b* = 1.25 and *c* = 0.3. The functional form and parameters of this target response profile were chosen heuristically to ensure that a) highest firing rates on the diagonal are favoured, b) the “punishment” of off-diagonal responses increases gradually with the distance from the diagonal for ratios close to the diagonal and c) responses far off the diagonal are equally and strongly “punished” in the cost function.

Calculating the spike density function (SDF, defined as *f*(*t*) = ∑exp−(*t*−*t_i_*)^2^/σ^2^, where *t_i_* is the time of the *i*th spike and *σ* = 400 ms.) for the PN at the midpoint of each stimulus presentation, we obtained a 10×10 response matrix ***r*** for all combinations of input rates *λ_i_*. A trial cost function *s*(***r***) is then defined as the “convolution” of the response matrix with a target 10×10 matrix ***r***: *s*(***r***) = −Σ*_i_*_,*j*_ ***r*** *_ij_* ***r*** *_ij_* (fig. 2). A smaller cost function indicates a “better” response profile. Using this multiplicative approach, rather than, for example, a Euclidean distance of profiles, avoids penalising upward deviations from the target profile on the diagonal. The target profile ***r*** was chosen heuristically (see fig 2 and its caption). We validated the choice of the target profile by evaluating the cost function for a number of observed response profiles and verifying that the ranking according to the cost function coincided with a manual assessment of the quality of the response profiles.

We optimised the performance of the model with respect to the cost function by adjusting the synaptic strength on the following connections: ORNs to specialist LNs; ORNs to generalist LN; and on all interconnections between LNs. The optimal set of parameters was determined by simulated annealing [42].

We simulated the model behaviour with target component ratios of 1∶1 for ORN-LN convergence rates of 200, 500 and 1000, for both conductance and rate based versions. For target component ratios 1∶3 and 1∶9, we used only the conductance-based version with an ORN-LN convergence rate of 1000.

#### Decision latency

Besides recognition accuracy assessed by the cost function detailed above, another measure of model performance is the decision latency. We define the latter as the time between stimulus onset and the first spike that is part of a stable, unbroken spike train, *i.e.*, the time from stimulus onset until the system reaches a steady state, irrespective of whether the response occurs to the target stimulus ratio (correctly) or not. We calculate decision latencies on the level of LNs as well as of the PN.

### Cell population models

In the minimal model we use only one LN per competing group (loosely identified as a ‘glomerulus’). As already mentioned in the introduction, in this model, the competition between LNs is masked by the effect of the first spike. In our rate-based approximation, such phasic phenomena are absent, and as we will see, the recognition of pheromone component ratios is more reliable. However, as insightful as the rate-based approach may be from a theoretical standpoint, a full conductance-based, spiking model is more convincing in order to elicit and assess the real potential of competition based ratio recognition systems.

We, therefore, built two more realistic models based on our minimal unit: (a) ‘*stacked model*’ where ratio recognition units are independent from each other, only connecting at the level of LNi, which now receives convergent input from ten generalist LNs; and (b) ‘*grouped model*’, where all LNs of the same type are organised into groups (‘glomeruli’) with all-to-all inter-LN connections between glomeruli. In both cases, respective connection strengths were downscaled by a factor of 10, while the amount of ORN stimulation to the LNs was kept the same as in the single-unit experiments.

## Results

### Minimalist model

We initially simulated the minimal model depicted in fig. 1 with all combinations of 10 different ORN firing rates in the two ORN populations and observed the response of the PN. In these simulations we used synapses with neurotransmitter release rate constant *α* = (20 ms)^−1^ and decay rate *β* = (50 ms)^−1^. After adjusting the ORN to LNa,b,c connections appropriately (*g*_ORN-LNsp_ roughly twice as strong as g_ORN-LNgen_), the target ratio of 1∶1 in the input rates elicits a reliable response in the PN neuron for a wide range of parameters for inter-LN synapses. However, we quite frequently also observed PN activation for non-target input ratios which could not be avoided by adjusting the parameters of the model by hand.

In an attempt to find an optimal set of parameters, we then used simulated annealing, with a cost function that rewarded PN activation for a 1∶1 target ratio and punished PN activation for all other input ratios (see Methods). To minimise non-deterministic effects of the Poissonian input signals, the cost function was evaluated by averaging over three trials for each input concentration ratio. All synaptic connection strengths except the output disinhibition pathway were subject to change in this procedure (*g*_syn_ of ORN-LNsp, ORN-LNgen, LNsp-LNgen, LNgen-LNsp, and LNsp-LNsp). Note that because of the symmetric pheromone target ratio of 1∶1, we fix *g*_ORNa-LNa_ = *g*_ORNb-LNb_ = *g*_ORN-LNsp_ and *g*_ORNa-LNc_ = *g*_ORNb-LNc_ = *g*_ORN-LNgen_.

In the following, inter-LN connection strengths are abbreviated as, respectively, *g*_LNi_ = *g*_LNsp-LNgen_, *g*_LNo_ = *g*_LNgen-LNsp_ and *g*_LNt_ = *g*_LNsp-LNsp_ (for *inbound*, *outbound* and *transverse*) and we assume symmetry of connection strengths between *a* and *b* populations again based on the 1∶1 symmetric target ratio. We tested three models with ORN-LN convergence ratios 200∶1, 500∶1 and 1000∶1 and both the full Hodgkin–Huxley model and an equivalent rate model (see Methods and Text S1) were used. For each model up to 40,000 iterations of the simplex annealing have been completed. The algorithm was terminated when the initial simplex of parameter vectors has shrunk to 0.01% of its initial size. The resulting best parameters found are shown in Table 1.

**Table 1:** Resulting parameters of the simplex annealing of the 1∶1 ratio recognition, for the various ORN-LN convergence rates.

Parameter	Conductance-based			Rate-based		
	200	500	1000	200	500	1000
*g*_ORN-LNsp_ [µS]	0.023	0.094	0.103	9.47·10^−3^	0.086	0.034
*g*_ORN-LNgen_ [µS]	0.013	0.072	0.074	6.06·10^−3^	0.045	0.021
*g*_LNi_ [µS]	0.185	0.490	0.499	0.146	0.153	0.098
*g*_LNt_ [µS]	0.185	0.500	0.389	0.081	0.100	0.187
*g*_LNo_ [µS]	0.184	0.347	0.386	0.076	0.181	0.099
Cost function	−1092	−1081	−1110	−2.65	−3.20	−3.69

The synaptic strengths are in µS.

**Figure 3:**
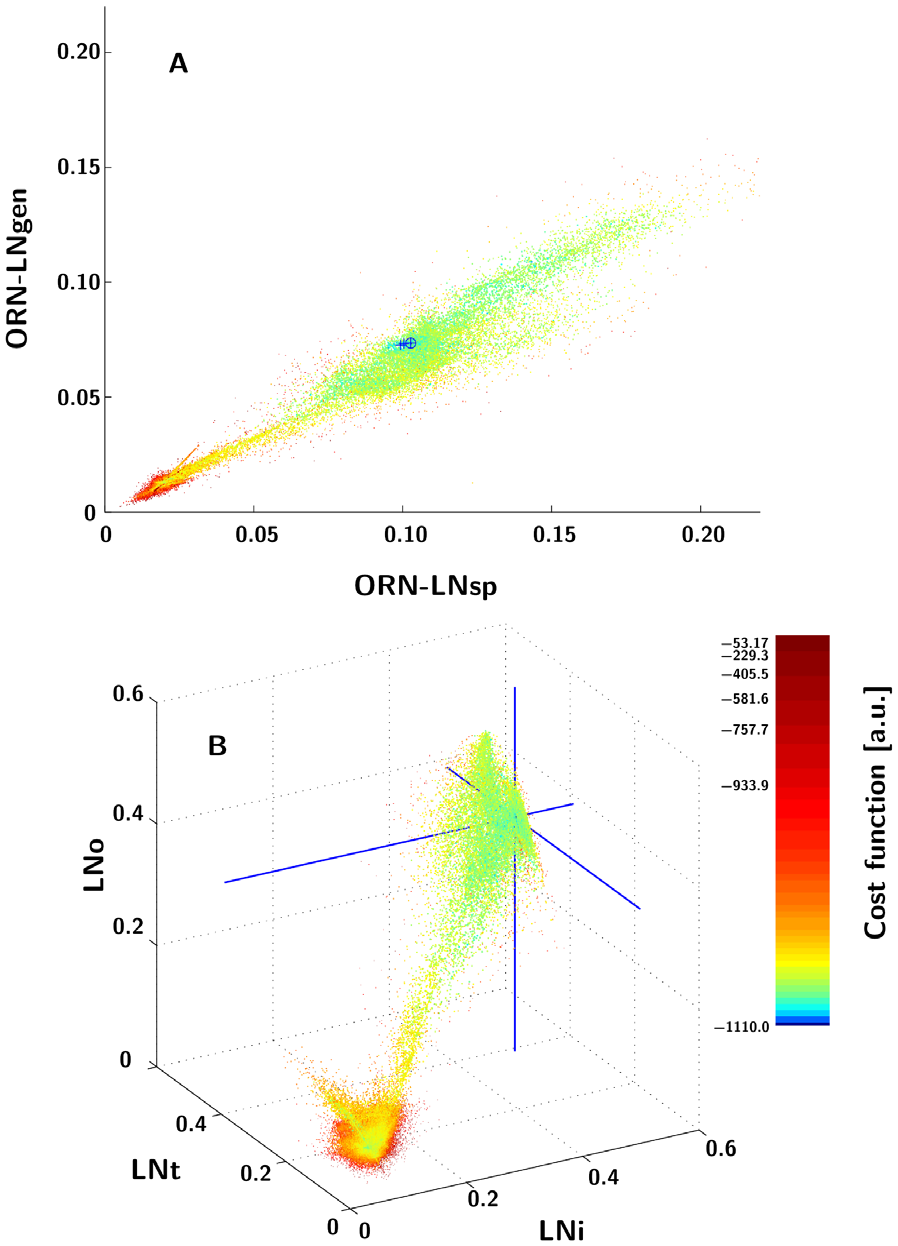
The cloud of data points (with cost function <0, *n* = 159,180) obtained in the simplex annealing of a basic conductance-based model with an ORN∶LN convergence rate of 1000. The colour of data points corresponds to the cost function value observed (see color bar). In each subfigure, the point with the best cost function is marked with a cross.

The model performance with respect to parameter changes as revealed in the course of annealing, is illustrated in fig 3. Low cost function parameter sets (good solutions) are coloured green to blue whereas worse solutions are in red colours (see colour bar and not the logarithmic scale). There are some very clear features in both the projection to the parameter space of ORN-LN connection strengths (panel A) and of the LN-LN connection strengths (panel B).

#### ORN-LN connection strengths

Obviously, there is a trade-off between having a reliable PN response for 1∶1 ratios (large enough *g*_ORN-LNgen_) and spurious PN activation for close by ratios (too large *g*_ORN-LNgen_). Naively, we expected *g*_ORN-LNsp_ = *2 g*_ORN-LNgen_. The best performing parameter set in the simulated annealing appears to have a somewhat larger *g*_ORN-LNgen_, with the ratio being approximately 1.7–1.8 ORNsp∶ORNgen (Table 1, Fig. 3A). This may surprise on first sight but it is perfectly reasonable as at equality of ORNa and ORNb firing, the generalist neuron has to have *stronger* input than the specialists, not only equal input. This bias must be enough for reliable LNgen responses with equal input but weak enough to have LNsp take over for the first off-diagonal terms (1∶1.3 and 1.3∶1 ratios). The region of successful parameter combinations of *g*_ORN-LNsp_ and *g*_ORN-LNgen_ is very well constrained indicating that the correct ratio between these conductances is very important for the correct recognition of pheromone component concentration ratio.

#### Inter-LN connection strengths

In contrast to the ORN-LN connections, our results do not indicate any clear rule for the inter-LN connection strengths other than that they have to be of approximately the same order of magnitude and not too weak overall. In this case, the “cloud” of appropriate synaptic strengths is more wide-spread (fig. 3B), although still well-delineated, with successful systems having an approximate ratio of 1∶1∶1 (LNi∶LNt∶LNo). Notably, there is a “side-arm” of parameter combinations for small *g*_LNo_ and *g*_LNi_ with strongly varying values for the strength of *g*_LNt_, indicating that the value of *g*_LNt_ is not essential for success in this area of parameter space. At close view, the cost function appears to be considerably scattered in repeated trials with the same or very close parameters due to the random nature of the Poissonian input, i.e., points with high cost function (red) can be very close to points with very low cost (green/blue) depending on the instantiations of the Poisson processes describing the ORNs. Even though we minimized the variability of the estimated cost function by taking averages over three trials for each parameter set, some of this uncertainty remains.

We note that this result indicates that while the existence of inter-LN competition is essential for ratio recognition, the exact details of it do not seem to matter. This is an interesting results at it seems to imply that no costly detailed genetic specification of inter-LN connectivity is necessary to achieve a functional MGC.

#### The balance of the inter-LN synaptic strengths

**Figure 4:**
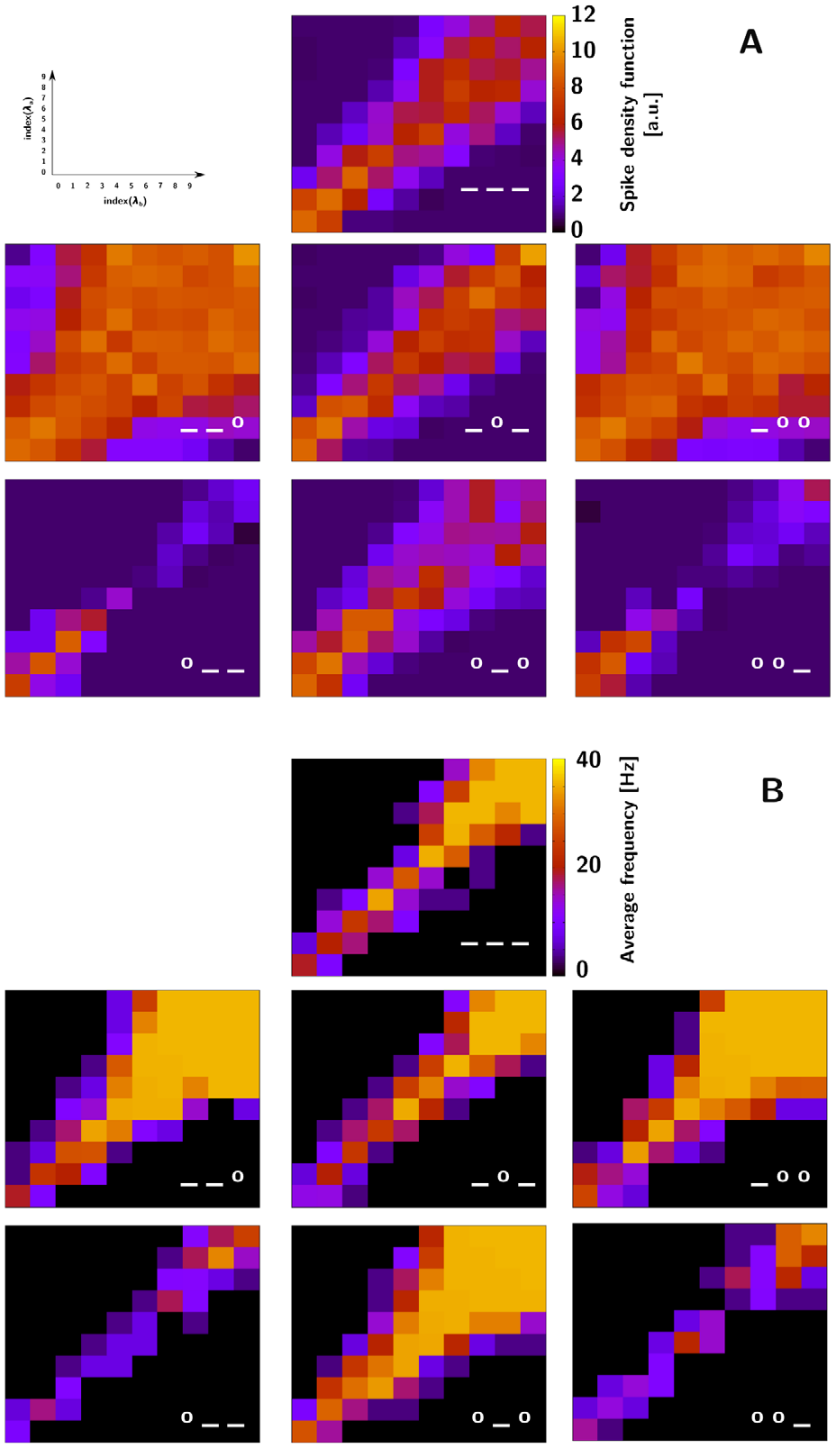
The balance of inter-LN connection strengths affects the ratio recognition accuracy. A) Response profiles in the conductance-based setup. B) Response profiles in the rate-based setup. The three symbols in each panel indicate, in order, the synaptic strengths on LNsp-LNgen, LNsp-LNsp and LNgen-LNsp connections, where “**_**” is the value for that connection found in the annealing optimisation (Table 1), and “**o**” is that value times 1.3 (A) or 1.5 (B). The *x*- and *y*-axes represent the concentration of individual components in the blend as the log of the Poisson firing rate of ORNs; the colour code of the squares shows the SDF (spike-density function) (A) and the average spiking rate (B), see respective colour bars. ORN-LN convergence rate used was 1000.

To elucidate the effect of inter-LN connections further, we obtained simulation results from a combination of discrete inter-LN *g* values set apart by a factor of 1.5 from the optimal solution as shown in fig. 4. The most detrimental change is caused by breaking the equality of *g*_LNi_ and its reciprocal *g*_LNo_, resulting in the loss of ratio selectivity towards higher pheromone component concentrations if *g*_LNi_<*g*_LNo_ and partial loss of response if *g*_LNi_>*g*_LNo_ *and g*_LNi_>*g*_LNt_. Most interestingly, given *g*_LNi_ = *g*_LNo_, the value of *g*_LNt_ does not seem to affect the ratio recognition across the entire concentration range, as can be seen in the fact that the case where g_LNi_ and g_LNo_ are elevated (“**o_o**”) and the case where g_LNt_ is elevated (“**_o_**”) are virtually identical despite having different *g*_LNt_. This agrees well with the observation of the extension of the cloud of good solutions in fig. 3 towards many different *g*_LNt_ values. The rate-based model (fig. 4B) behaved similarly with respect to these observations.

In a repeat of the above tests with baseline inter-LN conductances all set to 0.18 µS (rather than to the optimal values found in the annealing procedure (Table 2)), this baseline case still had the best cost function of all combinations studied (data not shown).

**Table 2:** Decision latencies measured at the PN, correlated (Pearson's product-moment) with the cost function, in a grouped arrangement of 10 LNs per glomerulus.

Case		Cost Function	PN	
*α* [ms^−1^]	*β* [ms^−1^]		Latency [ms]	corr. coeff.
0.050	0.025	−1145±51.8	137.4±4.19	−0.18*
0.042	0.025	−1096±47.1	131.2±4.20	−0.27†
0.060	0.025	−1182±57.3	139.1±3.97	−0.37†
0.050	0.021	−1180±42.5	141.5±4.55	−0.32†
0.050	0.030	−1084±48.3	131.4±3.94	−0.29†

Measurements were taken for different combinations of neurotransmitter release rise rate *α* and decay rate *β* (shown in ms^−1^) on inter-LN synapses. Higher cost function means better response. *n* = 120 for the case of baseline *α*−*β* combination, *n* = 100 for all other cases; values ±SD; (*) marks significance at *p* = 0.05 level, (†), at *p* = 0.01 level, n.s. means the *p* value is greater than 0.05.

We observed a surprising number of false positive responses, in particular towards higher total concentrations where the system responded even though the correct input ratio was not present. As we are convinced that the solutions found are optimal within the given architecture and constraints on parameters, we must assume that there are reasons why in principle a more specific response cannot be achieved. In order to uncover these underlying reasons, we undertook a closer analysis of the cases in which the system responded incorrectly to its input.

### Estimation of the probability of errors in ratio detection

#### “Spurious spikes”

Close analysis of the raw data revealed that the spurious activations for non-target ratio inputs are due to the excitation from both ORN populations producing the first spike in the generalist LN (LNc) before the excitation from the more active population (*e.g.*, ORNb) can activate its specialist LN (LNb). This occurs even though the rate of the ORNb population is higher than the rate of the ORNa population and thus the input to LNb should be stronger than the combined ORNa+ORNb input to LNc. The “spurious spike” in LNc will then cause a surge of inhibition onto the specialist LNs. Due to the time scales of the synapses, and the minimal value for the inter-LN conductances that allows winner-take-all dynamics, the outcome of the competition between LNs turns out to be determined largely by the first spike, *i.e.*, it is a competition in the latency to the first spike. Only in rare cases can the specialist LN overcome a spurious initial activation of LNc. As a result, LNs are locked into the wrong state for a significant part of or the entire time of stimulus presentation, and a wrong signal is sent downstream.

The incidence of spurious spikes depends on the properties of the Poisson spike trains arriving from the two ORN populations, in particular the firing rates *λ*_ORNa_ and *λ*_ORNb_ of the ORNs of each type and the number of ORNs converging onto each LN, as well as on the input integration in the LNs. The higher the overall population input rate to each LN, the lower is the variability of the summed input and hence, the probability of spurious spikes.

**Figure 5:**
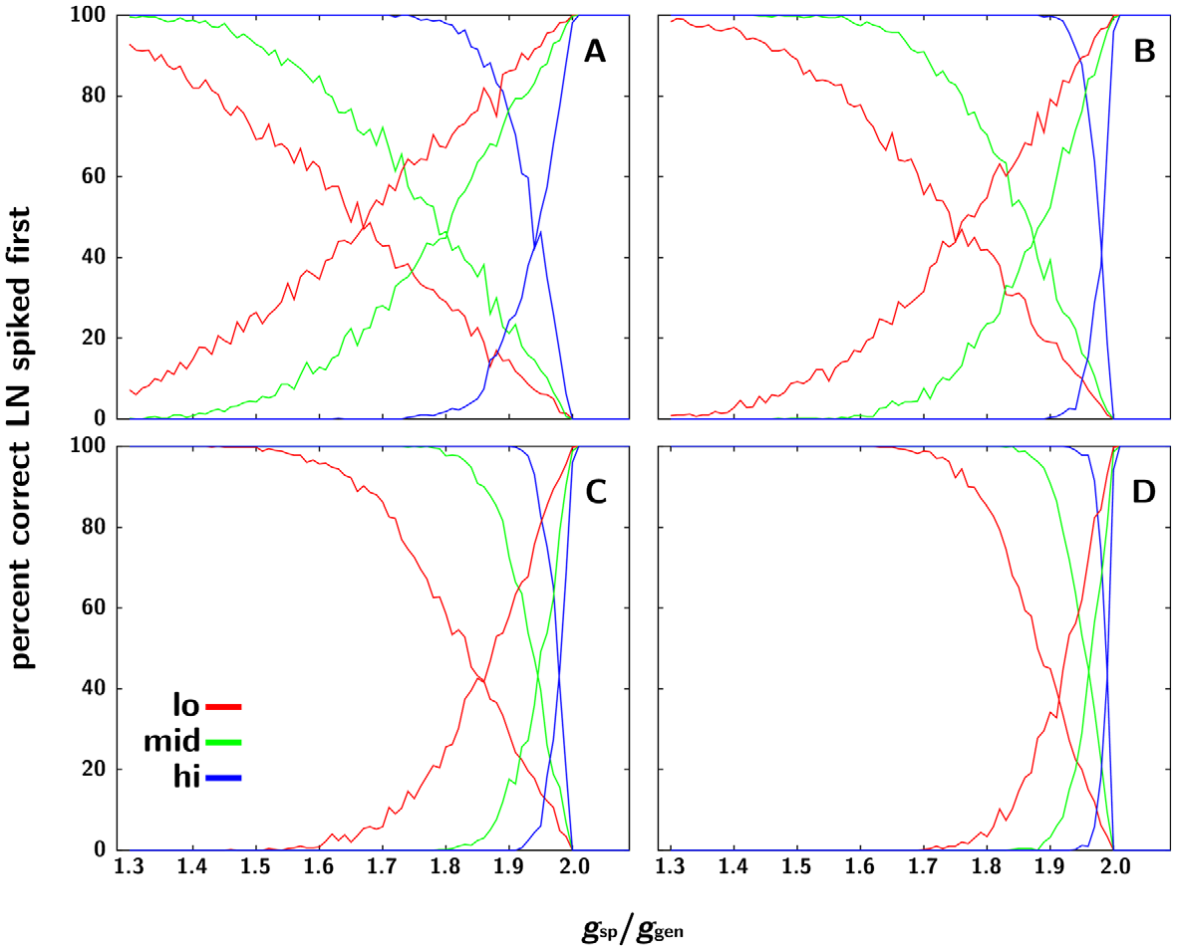
Percentage of LNc responses as a function of the ratio *g*_ORN-LNsp_/*g*_ORN-LNgen_. **A–D**, ORN∶LN convergence rates of 100, 200, 500 and 1000. The descending lines in each panel represent the percentage of responses to the 1∶1 ratio where LNc spikes first (true positives for 1∶1); ascending lines, the percentage of responses where an LNsp (specifically, LNb) fired first (true positives for non-1∶1 ratios immediately adjacent to the target 1∶1 ratio). The colours correspond to low (red), medium (green) and high (blue) pheromone blend concentrations. Note that there is no value of *g*_ORN-LNsp_/*g*_ORN-LNgen_ where both true positives are high simultaneously.

To focus on the LN latency based on input and cellular properties, we removed all inter-LN connections and used a simplified protocol including only the 1∶1 stimulus ratios and the ratio immediately adjacent to 1∶1, i.e. 1∶1.3, and estimated numerically the incidence of LN activations for several ORN-LN convergence rates, in the low, medium and high portions of the stimulus intensity, as shown in fig. 5. The lines descending as a function of the ratio *g*_ORN-LNsp_/*g*_ORN-LNgen_ are the probability of LNc responses to the target 1∶1 ratio (true positives for 1∶1) and the ascending lines are the responses of LNa/LNb to the non-target ratios (true positives for 1∶1.3). There are three separate lines for three different exemplary overall concentrations, low (red), medium (green) and high (blue). Optimally, we would expect to find *g*_ORN-LNsp_/*g*_ORN-LNgen_ such that true positives in both cases are (close to) 100%. However, there is no region in the plots where both percentages are high, not even for a single concentration value let alone for all three concentrations simultaneously. This convinced us that the model with single LNs as functional units, due to it being reduced to a latency-to-first-spike system, can on principle not perform to a better accuracy. We, therefore, extended our models to population models with the rationale that populations of neurons would be able to respond more gradually such that the latency to first spike does not dominate the competition. Alternatively, a population of the single-cell units could lead to a more informed response due to a second summation and thresholding process at the LNi, similar to boosting in machine learning.

### Extended models

**Figure 6:**
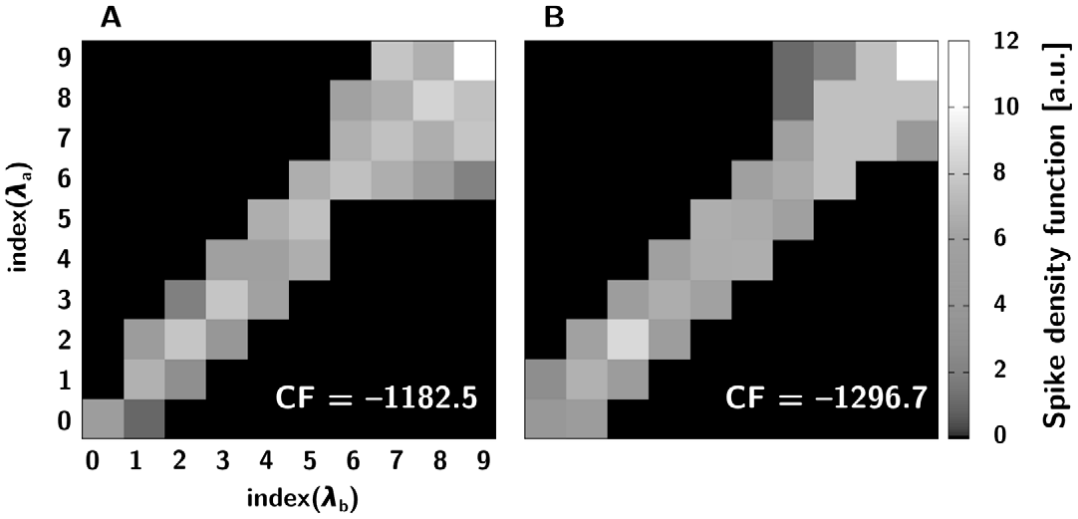
Response profiles for the population models. The grey scale plots show the spike density function of the PN in response to the different input frequencies in the stacked arrangement (A) and the grouped arrangement (B). These are the best outcomes of a total of 120 trials.Figure 7. Per-stimulus pair decision latencies (in ms) observed at the individual LNs, averaged over 120 trials (**A**, stacked arrangement, **B**, grouped arrangement).

We have taken a total of 100 repeated measurements of the performance in the two types of extended models using two arrangements of the elementary ratio recognition unit: stacked (multiple copies of the basic unit circuit converge onto LNi) and grouped (each LN is replaced by a population of like copies). The best outcomes of this test are shown in fig. 6.

In order to identify the character of the competition process we introduced and analysed the measure of decision latency, defined as the time until the system reaches a form of steady state in response to a rectangular onset of a constant odour stimulus (see methods for details). Specifically, where a neuron did not produce a spike train, or produced one that did not last to the end of the stimulus presentation, no data point was taken. Similarly, when spike trains persisted at LNs from more than a single LN group during the entire stimulus presentation (thus producing multiple effective winners), such data points were also omitted. Many (four to six) incidents of such multiple winners systematically appeared in every trial in the stacked case, which is not surprising as the constituent elementary units are completely isolated. In the grouped case, multiple winners were less frequent.

**Figure 7:**
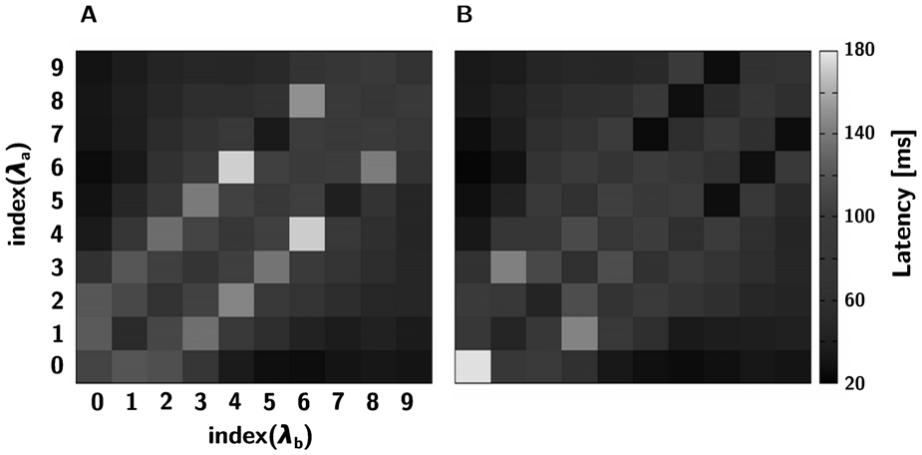
Decision latencies of LNs. The grey scale plots show the decision latency (in ms) per stimulus pair observed at the individual LNs, averaged over 120 trials. A) stacked arrangement. B) grouped arrangement.

The results of the latency measurements are illustrated in figure 7. As expected, the difficult off-diagonal ratios lead to the longest latencies followed by the 1∶1 ratio at small concentrations. It is interesting that the latency depends quite conspicuously on concentration, unlike the final outcome that, predominantly, does not.

**Figure 8:**
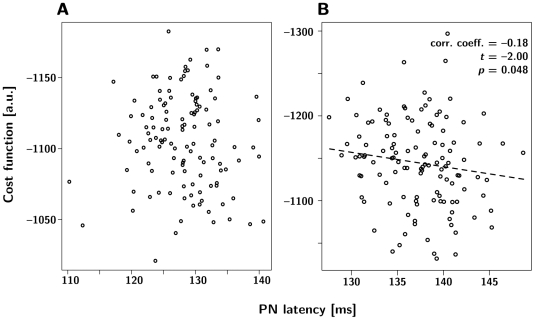
Correlation of average per-trial PN latency and the cost function in the extended models. (**A**, stacked arrangement, **B**, grouped arrangement).

To elucidate the relationship between the decision latency and the correct ratio recognition, we calculated the correlation between latency and correct recognition. We observed that in the grouped case, there is a significant *negative* correlation between latency and correctness, whereas we did not observe significant correlations in the stacked case (fig. 8). This came as a surprise because the intuition had been that a longer deliberation time (longer decision latency) should yield a better response. However, in this case the longer latency is caused by the problem being harder (the ratio being closer to 1∶1) which has the opposite effect on the response accuracy. Our simulation results indicate that in the trade-off between the two factors the second one appears to prevail.

Listed in table 2 are additional latency-accuracy correlations we have computed supporting this point of view. In the combination of higher *α* and lower *β* (which clearly means more stimulation overall), we see both a higher average cost function and stronger latency-cost function link. The former simply suggests our initial values for LN *α* and *β* were a little off the optimal mark.

The fact that decision latency for harder problems appears to be longer seems on first sight to contradict the results of [46] who found in bees that the behavioural decision latency for recognizing binary mixtures did not depend on the difficulty of the problem. However, the latencies observed in this study (∼700 ms) are almost an order of magnitude larger than the latencies in our model (∼100 ms) and observed during upwind flight in moths (∼150 ms, see [47]). We conclude that pheromone blend recognition in moths and recognition of binary mixtures in the general olfactory system of bees may not be directly comparable.

## Discussion

The simple model we propose represents an elementary ratio recognition unit, consisting of three LNs innervated by two groups of ORNs and connecting to a blend-specific PN through a disinhibition pathway. Of these units, in the moth Antennal Lobe there could be many, with variable connection strengths and hence, variable sensitivity and target ratio.

The pheromone communication in *Heliothis virescens* and an allied species *Helicoverpa zea* is an intricate case where a principal pheromone component, (Z)-11-hexadecenal (Z11–16:AL) is shared by both species, while two other compounds, (Z)-9-tetradecenal (Z9–14:AL) and (Z)-9-hexadecenal (Z9–16:AL) are, respectively, secondary for *H. virescens* and *H. zea*. In field studies, Vickers *et al* [43] reported fairly loose accuracy of component ratio recognition in *H. virescens* and *H. zea*. The authors distinguished four gradual behaviours, the most basic of which (taking flight) is enacted by a much broader pheromone component ratio than the ultimate behaviour (contacting the female). This lends support to our conception of multiple elementary units variously adapted, or ‘tuned,’ to detect the target pheromone component ratio. In this scenario, progressively greater populations of such units would be recruited as the strength of the signal increases and/or the component ratio approaches the target one.

Pheromone signalling has been shown [44] to sometimes involve an ‘antagonist’ component which disrupts the behaviour normally evoked by the ‘agonist’ pheromone components in the right proportion. Hansson et al [45], who studied the axonal arborisations of ORNs sensitive to the two agonist pheromone components in *H. virescens*, (Z)-9-tetradecenal (Z9–14:AL) and (Z)-11-hexadecenal (Z11–16:AL), found that they “do not display a clear-cut morphological separation into different glomeruli in the MGC” (denoted *a* and *b*) but rather innervate both, whereas the antagonist component's ORNs all terminate in a single glomerulus *c*. In this arrangement, the agonist and antagonist paths are independent from each other; the decision to trigger female-seeking behaviour is deferred to higher brain centres, where an agonist signal enables it unless overridden by the antagonist signal. In this case, our model would only cover the function of the agonist part.

Of the constraints on the synaptic strengths, we found that the ORN-LN connection strengths are much better constrained than the inter-LN connections, as evident from our cost function optimisation. Within the inter-LN connections it only seems to matter that g_LNi_ must be close to g_LNo_. If this requirement is met, the inter-specialist LN connection strength (LNt) may vary to a large extent, both upward of the LNi and LNo absolute value and downward. One probable interpretation of this result is that the competition between specialist LNs is not instrumental to the success of ratio recognition because the readout of the result is entirely based on the generalist LN population. If the two specialist populations fail to compete properly against each other but the competition of either with the generalist LNs is intact, the recognition of pheromone component ratios is likely almost unimpaired.

Our original intuition was that the MGC would achieve ratio recognition by straightforward competition in terms of the firing rate of populations of LNs which could have equivalently been expressed in a rate model. By considering the more detailed Hodgkin-Huxley model we found that the competition was realised in terms of latency to first spike rather than rate. One consequence of this latency coding in the system was a broadening of responses for higher overall pheromone concentrations which seems unintuitive from a pure rate perspective, because higher overall rates should have improved the signal to noise ratio. However, this broadening is observed experimentally (S. Anton, personal communication) supporting our results. Consequently, we feel that to advance the understanding of biological systems it is necessary to appreciate the way the account of a biological phenomenon may change depending on the type of model description.

## Supporting Information

Text S1 **The inference of the rate-based model of the Hodgkin–Huxley neuron.**
(PDF)
