# Supplementary material for: Competition-Based Model of Pheromone Component Ratio Detection in the Moth
Source: PLoS One. 2011 Feb 16;6(2):e16308. doi: 10.1371/journal.pone.0016308 (PMC3040183; doi:10.1371/journal.pone.0016308)
Supplement: Text S1 — The inference of the rate-based model of the Hodgkin–Huxley neuron. (PDF) [file pone.0016308.s001.pdf]

Supporting Materials for the PLoS paper *"Competition-based model of pheromone component ratio detection in the moth"* by Zavada *et al.*

Derivation of the rate-based model of Hodgkin–Huxley neurons with **αβ** *synapses*

The conductance-based Hodgkin–Huxley neuron model [1, 2] has become an essential instrument in computational neuroscience that allowed to simulate the time course of the membrane potential in response to stimulation, at arbitrary resolution. In larger-scale experiments, especially where the timescale of studied phenomena is much greater than the duration of a single spiking event, the precise form of the spike is of secondary concern. Instead, it proved to be convenient to represent the state of a neuron in terms of instantaneous spiking rate rather than its membrane potential.

In this section, following the analysis done by Nowotny & Rabinovich [3] for the Rall synapse [4], we set out to develop a rate-based representation of the Hodgkin–Huxley model specifically as used with the *αβ*-synapses.

The standard Hodgkin–Huxley model

In the general formulation, the classical Hodgkin–Huxley neuron model expresses the instantaneous rate of change of the membrane potential E as a dependency of its specific capacitance C and the total current passing through the membrane:

$$\frac{dE}{dt} = -\frac{1}{C}\sum I_i \tag{4}$$

where individual currents I_i include the sodium and potassium currents I_{Na} and I_{K}, a leak current I_{l}, the external current (such as that resulting from a neuromediator release at its dendrites) I_{ext}. The leak current is defined as $I_{\mathrm{l}} = g_{\mathrm{l}}(E - E_{\mathrm{l}})$, with a certain constant, voltage-independent leak conductance g_{l}. The currents I_{Na} and I_{K} are voltage-dependent, thus:

$$\begin{aligned} I_{\mathrm{Na}}(t) &= g_{\mathrm{Na}} m(t)^3 h(t)(E - E_{\mathrm{Na}}) \\ I_{\mathrm{K}}(t) &= g_{\mathrm{K}} n(t)^4 (E - E_{\mathrm{K}}) \end{aligned} \tag{5}$$

with each gating parameter $p = m$, n or h in (eq 5) defined by a first-order differential equation of the form:

$$\frac{dp}{dt} = \phi_p\big(E(t)\big)\,\big(1 - p(t)\big) - \chi_p\big(E(t)\big)\,p(t) \tag{6}$$

with these empirically found dependencies:

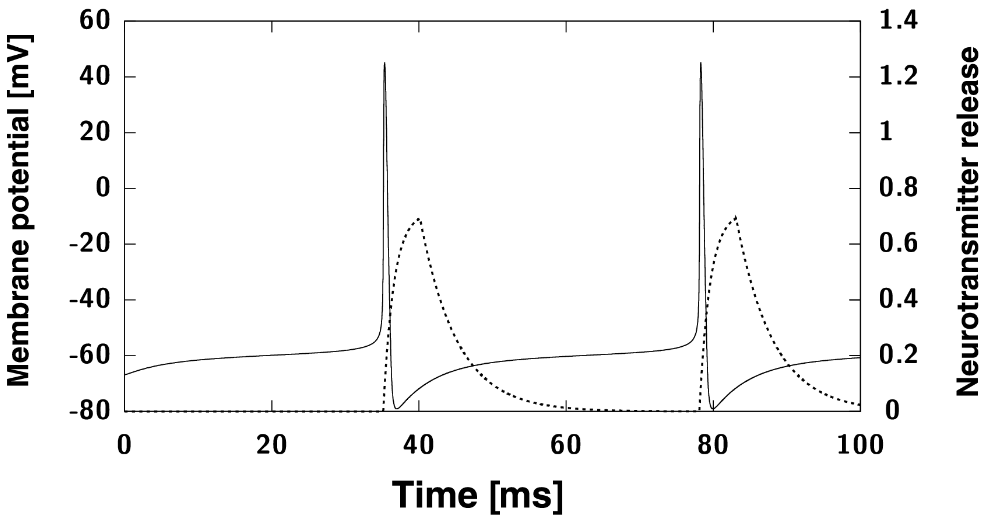

Figure S1: A Hodgkin–Huxley neuron (membrane potential E shown as solid line) starts continually spiking when injected a DC of 0.07 nA. Neurotransmitter release S at its axon is shown as the dotted line. The synapse is modelled with α = (20ms)$^{-1}$ and β = (50ms)$^{-1}$.

(7)
$$\begin{aligned}
\phi_n(E) &= 0.032\,(-50-E)/\Big(\exp\big((-50-E)/5\big)-1\Big)\\
\chi_n(E) &= 0.5\,\exp\big((-55-E)/40\big)\\
\phi_m(E) &= 0.32\,(-52-E)/\Big(\exp\big((-52-E)/4\big)-1\Big)\\
\chi_m(E) &= 0.28\,(25+E)/\Big(\exp\big((25+E)/5\big)-1\Big)\\
\phi_h(E) &= 0.128\,\exp\big((-48-E)/18\big)\\
\chi_h(E) &= 4/\Big(\exp\big((-25-E)/5\big)+1\Big)
\end{aligned}$$

In our study, we assumed C = 0.143 μF/cm^2, g_l = 0.0267 mS/cm^2, E_l = –63.563 mV, g_{Na} = 7.15 mS/cm^2, E_{Na} = 50 mV, g_K = 1.43 mS/cm^2, and E_K = –95 mV.

The αβ *synapse*

The $\alpha\beta$ synapse [5,6], as opposed to the synapse described by Rall [4], which has two separate equations for neurotransmitter release and binding, only models the amount of neurotransmitter acting on receptors of the post-synaptic membrane. The rate of change of active transmitter is governed by

(8)
$$\frac{dS}{dt} = \begin{cases} \alpha\,\big(1-S(t)\big) - \beta\,S(t) & \text{if } t - t_{\text{spike}} \le t_{\text{rel}},\\ -\beta\,S(t) & \text{otherwise}\end{cases}$$

where α and β are the rise and decay rates, respectively; the fixed parameter t_{rel} is the neurotransmitter release time, and t_{spike} is the time of the last spike in the pre-synaptic neuron. A synaptic current,

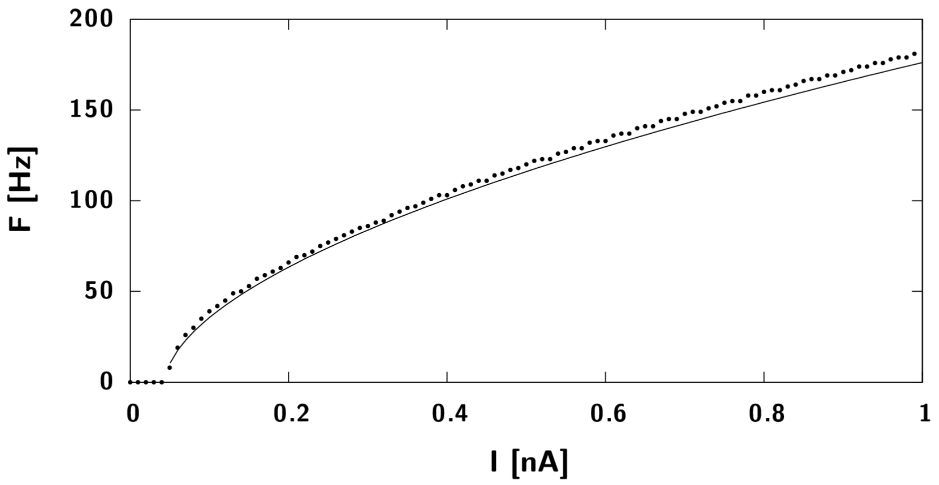

Figure S2: Firing rate F of a Hodgkin–Huxley neuron plotted as a function of its input current I (dots) and its empirical approximation given by eq. 10 (line).

$$I_{\mathrm{syn}} = g_{\mathrm{syn}}\, S\,(E_{\mathrm{pre}} - E_{\mathrm{syn}}), \tag{9}$$

where E_{pre} is the membrane potential of the neuron synapsed onto, and E_{syn}, the synapse reversal potential, is added to the sum of intrinsic currents of the post-synaptic neuron (eq. 4).

Figure S1 shows a Hodgkin–Huxley neuron which is caused to spike continually by a constant current injection, along with the changes in the amount of neurotransmitter released in its axon.

The rate-based model

In response to a constant input current I_{DC}, the HH neuron will spike tonically with a rate F that is a function of I_{DC}. The $F(I)$ curve for the HH neuron (fig. S2) can be fitted by an equation of the form

$$F = a\,(I_{\mathrm{syn}} - I_0)^r \tag{10}$$

where the empirically chosen parameters I_0 = 0.0439 nA, a = 0.185 and r = 0.564 [49].

Assuming that the time scale of a spike is much faster than the synaptic time scale, we can replace the presynaptic membrane potential E_{pre} in eq. 9 by a constant resting potential such that I_{syn} depends linearly on the amount of neurotransmitter S released for any given presynaptic frequency F. If we now determine $S(F)$, the conversion to a rate-based representation is complete.

The case of non-bounded increase of S

Let us consider a simpler case of eq. 8 (fig. S3) in which the rising part is independent of the current value of S (denoting release time as T):

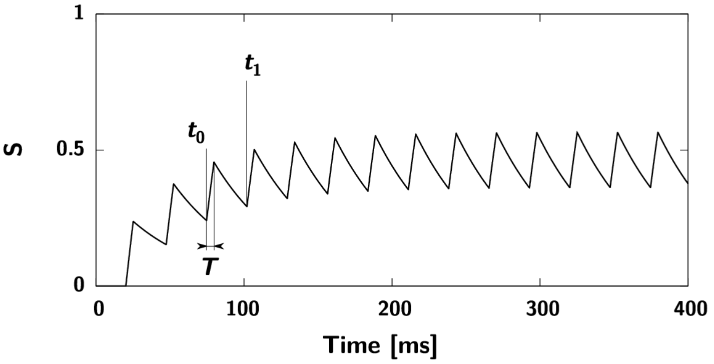

Figure S3: The course of the active transmitter concentration *S* as it stabilizes over time in response to tonic presynaptic spiking.

$$\frac{dS}{dt} = \begin{cases} \alpha - \beta\, S(t) & \text{if } t - t_{\text{spike}} \leq T, \\ -\beta\, S(t) & \text{otherwise} \end{cases} \tag{11}$$

which we now want to reduce to a single-equation form $dS/dt = -\beta S + \gamma$.

Let t_0 be a time where spike occurs and $t_1 = t_0 + 1/F$, and assume $1/F > T$ (fig. S3). Solving the differential equation for the rising part in (eq. 11) yields

$$\begin{aligned} S(t_0 + T) &= \frac{\alpha + \big(S(t_0)\,\beta - \alpha\big)\, e^{-\beta\, T}}{\beta} \\ &= \frac{\alpha\,(1 - e^{-\beta\, T}) + S(t_0)\,\beta\, e^{-\beta\, T}}{\beta} \\ &= \frac{\alpha}{\beta}\,(1 - e^{-\beta\, T}) + S(t_0)\, e^{-\beta\, T} \end{aligned} \tag{12}$$

And, for the decay part,

$$\begin{aligned} S(t_1) &= S(t_0 + T)\, e^{-\beta\,(\frac{1}{F} - T)} \\ &= \left(\frac{\alpha\,(1 - e^{-\beta\, T})}{\beta} + S(t_0)\, e^{-\beta\, T}\right) e^{-\beta\,(\frac{1}{F} - T)} \\ &= \frac{\alpha}{\beta}\,(1 - e^{-\beta\, T})\, e^{-\beta/F + \beta\, T} + S(t_0)\, e^{-\beta/F} \end{aligned} \tag{13}$$

Remembering that $S(t_1)$ can be reached in a single step via a similar function with γ, we can, after bringing the solution of $dS/dt = -\beta S + \gamma$ for $S(t)$ to the form similar to eq. 12, equate

$$S(t_1) = \frac{\gamma}{\beta}(1 - e^{-\beta/F}) + S(t_0)\, e^{-\beta/F} \tag{14}$$

Removing the common term $S(t_0)e^{-\beta/F}$ from (eqs 13 and 14) produces

$$(15) \qquad \frac{\alpha}{\beta}\,(1-e^{-\beta T})\,e^{-\beta/F+\beta T} = \frac{\gamma}{\beta}(1-e^{-\beta/F})$$

simplifying the left-hand side of which and solving for *γ* we get

$$(16) \qquad \gamma = \alpha\,\frac{e^{\beta T}-1}{e^{\beta/F}-1}$$

A list of all relevant parameters and variables used in the text is given in table S1.

Table S1. Parameters of the model and other relevant symbols used in the text, with descriptions.

**Parameter**	**Description**	**Value**
Olfactory receptor neurons (ORNs)		
λ_a	Firing rate of the ORNs of the first component	10 ... 200 Hz, in discrete increments per C_j (q.v.)
λ_b	Firing rate of the ORNs of the second component	10 ... 200 Hz
Local neurons (LN) and Projection neurons (PN)		
C	Membrane capacitance	0.143 μF/cm^2
g_l	Leak conductance	0.0267 mS/cm^2
E_l	Leak reversal potential	–63.563 mV
g_{Na}	Maximal sodium conductance	7.15 mS/cm^2
E_{Na}	Sodium reversal potential	50 mV
g_K	Maximal potassium conductance	1.43 mS/cm^2
E_K	Potassium reversal potential	–95 mV
LN Synapses		
$g_{ORN\text{-}LNsp}$	Conductance on the ORN-LNsp connection	See table 2
$g_{ORN\text{-}LNgen}$	Strength of the ORN-LNgen connection	“-“
g_{LNi}	Strength of the LNgen-LNsp connections	“-“
g_{LNt}	Strength of the inter-LNsp connections	“-“
g_{LNo}	Strength of the LNsp-LNgen connections	“-“
$t_{release}$	Duration of transmitter release	5ms
E_{syn}	Reversal potential	0mV
E_{pre}	Presynaptic threshold potential	–20mV
α	Rate of transmitter activation	(20ms)$^{-1}$
	Adjusted rate of transmitter activation for linearized synapse equations	(27.79ms)$^{-1}$
β	Rate of transmitter removal from the synaptic cleft	(50ms)$^{-1}$
Target cost function profile		
$C_{ij.}$	Multiplier for component concentration at *j*th step (for the cost function formula, see legend to fig. 2)	2×1.3^i, $j = 0 \dots 9$
b	Steepness of the target profile crest	1.25
a	Height of the target profile crest	18
c	Part of the crest submersed below 0	0.3

References

1. Hodgkin AL, Huxley AF, Katz B (1952) Measurement of current-voltage relations in the membrane of the giant axon of Loligo. *J Physiol* **116:** 424-448.
2. Traub RD, Miles R (1991) Neuronal networks of the hippocampus. Cambridge University Press. 304 p.
3. Nowotny T, Rabinovich MI (2007) Dynamical origin of independent spiking and bursting activity in neural microcircuits. *Phys Rev Lett* **98:** 128106.
4. Rall W (1967) Distinguishing theoretical synaptic potentials computed for different soma-dendritic distributions of synaptic input. *J Neurophysiol* **30:** 1138–1168.
5. Destexhe A, Mainen ZF, Sejnowski TJ (1994) Synthesis of models for excitable membranes, synaptic transmission and neuromodulation using a common kinetic formalism. *J Comp Neurosci* **1:** 195-230.
6. Destexhe A, Mainen ZF, Sejnowski TJ (1994) An efficient method for computing synaptic conductances based on a kinetic model of receptor binding. *Neural Comput* **6:** 14-18.
